# Visual load does not decrease the auditory steady‐state response to 40‐Hz amplitude‐modulated tones

**DOI:** 10.1111/psyp.13689

**Published:** 2020-09-18

**Authors:** Malina Szychowska, Stefan Wiens

**Affiliations:** ^1^ Gösta Ekman Laboratory Department of Psychology Stockholm University Stockholm Sweden

**Keywords:** crossmodal attention, early‐filter theory, EEG, envelope following response, task difficulty

## Abstract

The auditory pathway consists of multiple recurrent loops of afferent and efferent connections that extend from the cochlea up to the prefrontal cortex. The early‐filter theory proposes that these loops allow top‐down filtering of early and middle latency auditory responses. Furthermore, the adaptive filtering model suggests that the filtering of irrelevant auditory stimuli should start lower in the pathway during more demanding tasks. If so, the 40‐Hz auditory steady‐state responses (ASSRs) to irrelevant sounds should be affected by top‐down crossmodal attention to a visual task, and effects should vary with the load of the visual task. Because few studies have examined this possibility, we conducted two preregistered studies that manipulated visual load (Study 1: *N* = 43, Study 2: *N* = 45). Study 1 used two levels (low and high), and Study 2 used four levels (no, low, high, and very high). Subjects were asked to ignore a 500‐Hz task‐irrelevant tone that was amplitude‐modulated to evoke 40‐Hz ASSRs. Results from Bayesian analyses provided moderate to extreme support for no effect of load (or of a task) on ASSRs. Results also supported no interaction with time (i.e., over blocks, over minutes, or with changes in ASSRs that were synchronized with the onset of the visual stimuli). Further, results provided moderate support for no correlation between the effects of load and working memory capacity. Because the present findings support the robustness of ASSRs against manipulations of crossmodal attention, they are not consistent with the adaptive filtering model.

## Abstract 2

The adaptive filtering model suggests that the filtering of irrelevant auditory stimuli should start lower in the auditory pathway during more demanding tasks. Two preregistered studies (*N* = 43, *N* = 45) examined the effects of visual perceptual load (from no to very high) on the 40‐Hz auditory steady‐state response (ASSR) to a task‐irrelevant tone. Bayesian analyses provided evidence for no effect of load. This robustness of ASSR against manipulations of crossmodal attention is not consistent with the adaptive filter model.

## 1 INTRODUCTION

Hearing is an early‐warning system that constantly monitors the environment (Henneman, 1952). Unlike vision, hearing is not limited in scope and can detect stimuli from any direction. However, some auditory inputs are task‐irrelevant and potentially distracting. Accordingly, although hearing is useful as a warning system, the ability to dampen the processing of auditory distractors seems highly relevant for daily tasks, such as when one is working in an open‐space office and writing a report while other people are talking on their phones. Therefore, a balance is needed between stimulus‐driven (early‐warning, bottom‐up) and goal‐oriented (top‐down) attention (Corbetta, Patel, & Shulman, 2008).

To investigate this balance, research has used crossmodal attention tasks to study the extent to which goal‐oriented attention to a visual task dampens or filters the processing of task‐irrelevant sounds. According to early‐filter theory, the auditory pathway consists of loops of afferent and efferent connections (corticopetal‐corticofugal loops) that reach all the way down to the cochlea and allow top‐down attentional modulation of very early as well as late auditory responses by the prefrontal cortex (Marsh & Campbell, 2016; Sarter, Gehring, & Kozak, 2006) and by other, multisensory areas (Driver & Noesselt, 2008; Macaluso & Driver, 2005). Also, selection is believed to be more efficient when the ignored stimuli are predictable (Marsh & Campbell, 2016). Furthermore, according to the adaptive filtering model, the filtering is dynamic; thus, the modulation of the responses in the auditory pathway should occur at earlier stages of processing for difficult tasks than for easy tasks (Giard, Fort, Mouchetant‐Rostaing, & Pernier, 2000). Taken together, these models suggest that effects of crossmodal attention to a visual task on the processing of task‐irrelevant, predictable sounds vary with the visual task demand and can be detected at various stages of auditory processing.

Auditory processing at middle latency can be conveniently studied with auditory steady‐state responses (ASSRs). Typically, ASSRs are recorded with electroencephalography (EEG) and are elicited by trains of clicks or noise bursts (Galambos, Makeig, & Talmachoff, 1981), sounds that are modulated in amplitude or frequency (Picton, Skinner, Champagne, Kellett, & Maiste, 1987), or other periodic, rhythmic stimuli (for review, see Picton, John, Dimitrijevic, & Purcell, 2003). Because the stimuli that evoke ASSRs are highly predictable, ASSRs should be sensitive to crossmodal attention effects on auditory processing, as implied by Marsh and Campbell (2016).

Furthermore, because ASSRs do not require subjective responses, they are widely used in audiology to measure the auditory threshold of individuals who cannot provide a subjective response (e.g., preverbal children) (Dimitrijevic et al., 2002; Kuwada, Batra, & Maher, 1986; Lins et al., 1996; Picton et al., 2003). However, because these individuals can potentially ignore the auditory stimuli and focus their attention elsewhere, it is relevant for this clinical context to investigate the extent to which ASSRs are sensitive to changes in crossmodal attention.

The most popular method to evoke ASSRs is with amplitude‐modulated tones. For example, the amplitude of a 500‐Hz tone can be modulated at 40 Hz with 100% modulation depth; that is, the amplitude of this tone changes from zero to maximum and back to zero 40 times per second (i.e., every 25 ms). According to the *superposition hypothesis* (Bohórquez & Özdamar, 2008; Galambos et al., 1981), each tone peak elicits an event‐related potential (ERP) that comprises a series of positive and negative amplitude deflections. As the tone peaks are repeated, the ERPs to these consecutive tone peaks overlap and sum to a periodic, steady‐state brain response. The frequency of this response is equal to the amplitude‐modulation frequency of the stimulus. Thus, for a tone that is amplitude‐modulated at 40 Hz, the steady‐state response occurs at 40 Hz (Collura, 1995; Gutschalk et al., 1999; Herdman et al., 2002).

ASSRs are strongest and most commonly measured at 40 Hz (Galambos et al., 1981; Hari, Hämäläinen, & Joutsiniemi, 1989; Picton et al., 1987; Roß, Borgmann, Draganova, Roberts, & Pantev, 2000). Evidence suggests that for 40‐Hz ASSRs, the overlap during superposition includes mainly ERP peaks from wave V, Pa, Na, Pb, and Nb (Bohórquez & Özdamar, 2008; Galambos et al., 1981; but only Pa, Na, Pb, and Nb in Gutschalk et al., 1999). Because these peaks are separated in time by approximately 25 ms, which is the same interval as that between consecutive cycles in a 40‐Hz stimulus, this overlap explains why 40‐Hz ASSRs are strongest (Picton et al., 2003). Research shows that the wave V is generated in the brainstem, in the vicinity of the lateral lemniscus and inferior colliculus (Hall, 2007; Liveson & Ma, 1999; Marsh & Campbell, 2016; Møller & Jannetta, 1983), and the middle‐latency responses (MLRs: peaks Pa, Na, Pb, and Nb) are generated by the auditory cortex, specifically Heschl's gyrus (Godey, Schwartz, de Graaf, Chauvel, & Liégeois‐Chauvel, 2001; Kuriki, Nogai, & Hirata, 1995). Consistent with these findings, research shows that 40‐Hz ASSRs have generators in both the brainstem and the auditory cortex, specifically Heschl's gyrus (Engelien, Schulz, Ross, Arolt, & Pantev, 2000; Gutschalk et al., 1999; Herdman et al., 2002; Korczak, Smart, Delgado, Strobel, & Bradford, 2012; Luke, De Vos, & Wouters, 2017; Picton et al., 2003; Plourde, Stapells, & Picton, 1991).

However, brain activity is not linear, and in some studies, the recorded ASSRs did not match the simulated ERP overlap (Azzena et al., 1995; Ross, Herdman, & Pantev, 2005; Zhang, Peng, Zhang, & Hu, 2013). Therefore, there may be an additional mechanism of ASSRs in terms of independent activity of specific neurons responding at preferred modulation rates, thereby inducing entrainment of neuronal rhythm. This additional mechanism is postulated by the *oscillatory entrainment hypothesis* (Azzena et al., 1995; Pantev, Roberts, Elbert, Roβ, & Wienbruch, 1996; Picton et al., 2003; Ross et al., 2005; Zhang et al., 2013).

Regarding the effects of crossmodal attention on 40‐Hz ASSRs, most studies have compared ASSRs recorded during a visual task with those recorded during an auditory task (e.g., Ross, Picton, Herdman, & Pantev, 2004; Saupe, Widmann, Bendixen, Müller, & Schröger, 2009). However, because modality switching and task switching within a modality seem to operate by separate mechanisms (Hunt & Kingstone, 2004; Murray, Santis, Thut, & Wylie, 2009), it is unresolved whether any differences in the studies' findings reflect switching attention between modalities, between tasks, or both. To study the effects of crossmodal attention while minimizing the effects of modality switching, a preferable approach is to manipulate visual task difficulty or load while simultaneously presenting task‐irrelevant auditory stimuli.

Only a few previous studies have tested the effects of crossmodal attention on 40‐Hz ASSRs by manipulating the difficulty or load of the visual task. These visual tasks had several different load levels: detecting an easy versus difficult target (Parks, Hilimire, & Corballis, 2011), detecting versus discriminating a change in target brightness (de Jong, Toffanin, & Harbers, 2010), reading versus performing a visual search task (Griskova‐Bulanova, Ruksenas, Dapsys, Maciulis, & Arnfred, 2011), playing an easy versus difficult Tetris game (Roth et al., 2013), flying an airplane in a flight simulator during self‐reported low versus high mental workload (Tsuruhara, Arake, Ogawa, Aiba, & Tomitsuka, 2015), and performing *n*‐back tasks with different *n* to vary the difficulty (Yokota & Naruse, 2015; Yokota, Tanaka, Miyamoto, & Naruse, 2017).

It is unclear whether these studies provide evidence for an effect of visual load on 40‐Hz ASSRs. Four studies showed a statistically significant decrease in ASSRs as a result of visual load. Of these studies, three showed smaller ASSR amplitude for high load than for low load (Roth et al., 2013; Tsuruhara et al., 2015; Yokota & Naruse, 2015), and two showed lower phase locking for high than for low load (Yokota et al., 2017, Yokota & Naruse, 2015). However, in other studies, either the effect was unclear, or there was no apparent statistically significant effect. Specifically, a study by de Jong et al. (2010) showed no effect of load on ASSRs on expected electrodes but claimed (post hoc) a statistically significant effect on ASSR amplitude at unexpected (occipital) electrodes. Two other studies did not report statistically significant differences between the visual conditions (Griskova‐Bulanova et al., 2011; Parks et al., 2011). Finally, one study did not analyze the ASSRs, because the authors were interested in different responses (Mossbridge, Grabowecky, & Suzuki, 2013). Thus, these studies do not seem to provide clear evidence for or against an effect of visual load on ASSRs.

Furthermore, previous studies have several important limitations. First, several studies used visual stimuli that differed physically between the loads (Griskova‐Bulanova et al., 2011; Roth et al., 2013; Tsuruhara et al., 2015). Because visual conditions differed, any differences in ASSRs between loads could have been driven by these physical differences. Second, it is difficult to rule out flexibility in data processing in any of the studies. For example, several studies used a data‐driven method to select the electrodes for analysis (Griskova‐Bulanova et al., 2011; de Jong et al., 2010; Parks et al., 2011; Roth et al., 2013). Therefore, it is unclear whether results are biased (John, Loewenstein, & Prelec, 2012; Luck & Gaspelin, 2017; Simmons, Nelson, & Simonsohn, 2011). Third, statistically nonsignificant effects cannot be interpreted as direct support for no effect (Dienes, 2008; Wiens & Nilsson, 2017), and statistically significant effects cannot be interpreted as direct support for a theoretical effect (Dienes & McLatchie, 2018).

The main goal of the present research was to examine the effects of load on 40‐Hz ASSRs while avoiding the above‐mentioned limitations. In Study 1, we used a visual task with two levels of load, similar to that used by Parks et al. (2011). Subjects were asked to detect a target in a rapid series of crosses. On each trial, the cross was either upright or upside‐down, and it had one of the five colors. In *low* load, the target was a cross of a particular color but of either orientation (the two targets were red upright and red inverted crosses). In *high* load, the target was a combination of color and orientation (the two targets were yellow upright and green inverted crosses). Because high load presumably requires more attentional resources than low load, this task is considered to be a prototypical manipulation of *perceptual load* (Lavie, 2005). The visual and auditory stimuli in the two load conditions were physically identical and only the instructions differed, to ensure that any differences in the ASSRs were not confounded by physical differences in the load conditions.

In both studies, ASSRs were measured in terms of their mean amplitude and their intertrial phase coherence (ITC). We used both measures for two reasons. First, they reflect independent aspects of the response: Whereas amplitude measures the size of the response across trials, ITC measures the coherence of the phase among trials. It is possible that two conditions could differ in mean amplitude but not in ITC, or vice versa. For example, if high load has lower mean amplitude than low load, this difference could be driven by a lower ITC for high load than low load. Therefore, it is informative to measure both mean amplitude and ITC. Second, previous research has used various measures of amplitude and ITC, and it is unclear whether the choice of a particular measure was data‐driven (and thus potentially biased). Therefore, we preregistered to measure both amplitude and ITC and to capture the signal level (i.e., 40 Hz) relative to the noise level (i.e., neighboring frequencies).

In preview, Bayesian analyses in Study 1 showed no effect of visual load on the ASSRs. As a follow‐up, Study 2 used a similar visual task but included three load levels and a passive viewing condition (as baseline). Subjects were asked to detect target letters in a rapid series of letters that differed in name, color, and capitalization. In *no load*, there was no target, and subjects passively viewed the letter stream. The low load and high load conditions resembled those in Study 1: In *low load*, targets were letters of a particular color, regardless of name and capitalization (e.g., red). In *high load*, targets were letters of a combination of color and name, regardless of capitalization (e.g., yellow *K* or *k*, and blue *R* or *r*). In *very high load*, target letters were a combination of color, name, and capitalization (e.g., green *h* and purple *M*). In the different conditions, the visual stimuli were physically identical but occurred in different proportions (to retain a constant proportion of targets). Thus, any differences in the ASSRs were probably not due to physical differences in the load conditions.

To minimize bias in the results, we preregistered both studies with regard to the analyzed electrodes, the ASSR measurements, and the statistical analyses (Nosek, Ebersole, DeHaven, & Mellor, 2018). Also, we conducted Bayesian hypothesis tests (i.e., Bayesian one‐sample *t* tests) and computed the Bayes factor (*BF*) to differentiate among results that supported the null hypothesis, supported the alternative hypothesis, or were inconclusive (Dienes, 2016).

In both studies, we recorded discrimination ability and reaction times to targets to assess whether the load manipulations were strong enough. In addition, after finishing data collection for Study 1, we realized that we could conduct an exploratory analysis of the visual P3. The visual P3 was used as an additional measure of load manipulation to index increased attention to the visual targets versus nontargets (Polich, 2007). The visual P3 to targets versus nontargets should be smaller during high load than during low load because of reduced certainty about the target or increased allocation of cognitive resources to the task (Gomarus, Althaus, Wijers, & Minderaa, 2006; Hagen, Gatherwright, Lopez, & Polich, 2006; Kok, 2001; Watter, Geffen, & Geffen, 2001). Because results for the visual P3 for Study 1 were promising, we preregistered the analyses of the visual P3 for Study 2. As an additional manipulation check for load, Study 2 recorded subjective ratings of workload in terms of mental demand, physical demand, temporal demand, performance, effort, and frustration (Hart & Staveland, 1988).

A final goal of the present research was to investigate the relationship between working memory capacity (WMC) and the effect of visual load on ASSRs. No previous study has addressed this specific question, even though the new early‐filter model predicts a relationship (Marsh & Campbell, 2016). According to the model, the top‐down ability to control the early stages of auditory processing should be better in individuals with high WMC than in those with low WMC because high WMC is associated with greater attentional control. In support, a crossmodal study of auditory brainstem responses (ABRs) to task‐irrelevant auditory stimuli found that relative to individuals with low WMC, individuals with high WMC had smaller ABRs during high load than during low load in a visual n‐back task (Sörqvist, Stenfelt, & Rönnberg, 2012). Specifically, WMC was correlated with a greater decrease of the ABR from low to high load. Notably, during low load, the ABRs did not differ between individuals with high and low WMC.

However, results from a behavioral study suggest that WMC effects may not be straightforward (Halin, Marsh, & Sörqvist, 2015). In that study, subjects were administered a surprise memory test about the content of the background speech (auditory distractors) that they had been asked to ignore during a previous visual n‐back task at low and high load. Individuals with high WMC had low performance on the incidental memory test after both low and high load, whereas individuals with low WMC showed a decrease from low to high load. After low load, incidental memory performance was worse in individuals with high than with low WMC. These results suggest that whereas individuals with low WMC can effectively ignore task‐irrelevant sounds during high load only, individuals with high WMC may be able to ignore these sounds during both high and low load.

These two studies differed with regard to the directionality of the correlation between WMC and the effect of load (low vs. high) on the distractors. In one study (Sörqvist et al., 2012), high WMC was associated with a larger decrease in distractor processing from low to high load, whereas in the other study (Halin et al., 2015), high WMC was associated with a smaller decrease in distractor processing from low to high load. Because of these inconsistencies, we did not predict a specific direction of the correlation between WMC and the effect of visual load on ASSRs.

To summarize, we examined the effects of visual load on 40‐Hz ASSRs. Study 1 involved a prototypical visual load task with two levels of perceptual load. Study 2 involved a similar visual load task but with three levels of load and a passive viewing condition. As manipulation checks, we recorded task performance in the form of signal detection index *d′* and reaction time to targets (Studies 1 and 2), the visual P3 to targets versus nontargets (Studies 1 and 2), and subjective ratings of workload (Study 2). We also measured WMC to investigate the relationship between the effects of load on 40‐Hz ASSRs and WMC.

## 2 STUDY 1

### 2.1 Method

All Supporting Information is available at a university depository (Wiens & Szychowska, 2020). This material includes raw data, scripts, and results of main and supplementary analyses.

Method and analyses were preregistered before any data were collected (https://doi.org/10.17605/OSF.IO/UYJVA). Deviations from the preregistration are noted below. Analyses that were not mentioned in the preregistration are labeled as exploratory. Data were processed and analyzed with the MNE‐Python package (Gramfort et al., 2013) in Python (Van Rossum & Drake, 2009) and R (R Core Team, 2020) in RStudio (RStudio Team, 2019).

Forty‐four subjects were recruited from local universities and through online billboards in Stockholm, Sweden. One subject had to be excluded because of electrode failure. The final sample consisted of 43 subjects (age *M* = 25.65, *SD* = 4.37, 39 right‐handed, 23 male). Subjects were compensated with a cinema ticket, a 100‐SEK gift voucher, or course credits. Before the experiment, subjects provided written consent in accordance with the Declaration of Helsinki. The study was conducted in accordance with the principles of the regional ethics board. Subjects were recruited if they reported that they were between 18 and 40 years old, had no history of neurological or psychological illness, and had a normal or corrected‐to‐normal vision and normal hearing. To ensure normal hearing (≤20 dB HL), hearing was tested directly at the beginning of the experiment with pure tone audiometry at 500 Hz (the relevant frequency for the study), 750 Hz, and 1,000 Hz. Although we preregistered that data collection would finish by the end of May 2018, we continued collecting data after that date because the *BF* indicated inconclusive results (*BF* < 3). We analyzed the data after testing several new subjects, and we continued testing until we reached *BF* > 3.

During the preprocessing of the EEG data, five subjects retained less than 70% of the trials in at least one block after artifact rejection. In line with our preregistered criteria, these subjects were initially excluded; after exclusion, the sample consisted of 38 subjects (age *M* = 25.76, *SD* = 4.02, 34 right‐handed, 20 male). However, because results were comparable for the sample before and after exclusion (see Supporting Information), we decided to report only the results for the larger sample (*N* = 43).

### 2.2 Auditory stimuli

The auditory stimulus was an amplitude‐modulated tone with a carrier frequency (*f*_c_) of 500 Hz, a modulation frequency (*f*_m_) of 40.96 Hz, and a modulation depth of 100%. This particular modulation frequency was chosen because it was the closest frequency to the target frequency of 40 Hz that our EEG equipment could resolve (at a sampling frequency of 1,024 Hz, 1,024/40.96 = 25 cycles). To minimize the response to the carrier frequency, the carrier frequency started with either the condensation or the rarefaction phase. For each subject, the phase was assigned randomly for the first block and then alternated every two blocks (so that in a set of low and high load, phase was identical). Tones were presented binaurally at 60 dB SL through in‐ear tubephones (ER2; Etymotic Research Inc., IL; www.etymotic.com).

### 2.3 Procedure

#### 2.3.1 Visual detection task

**FIGURE 1:**
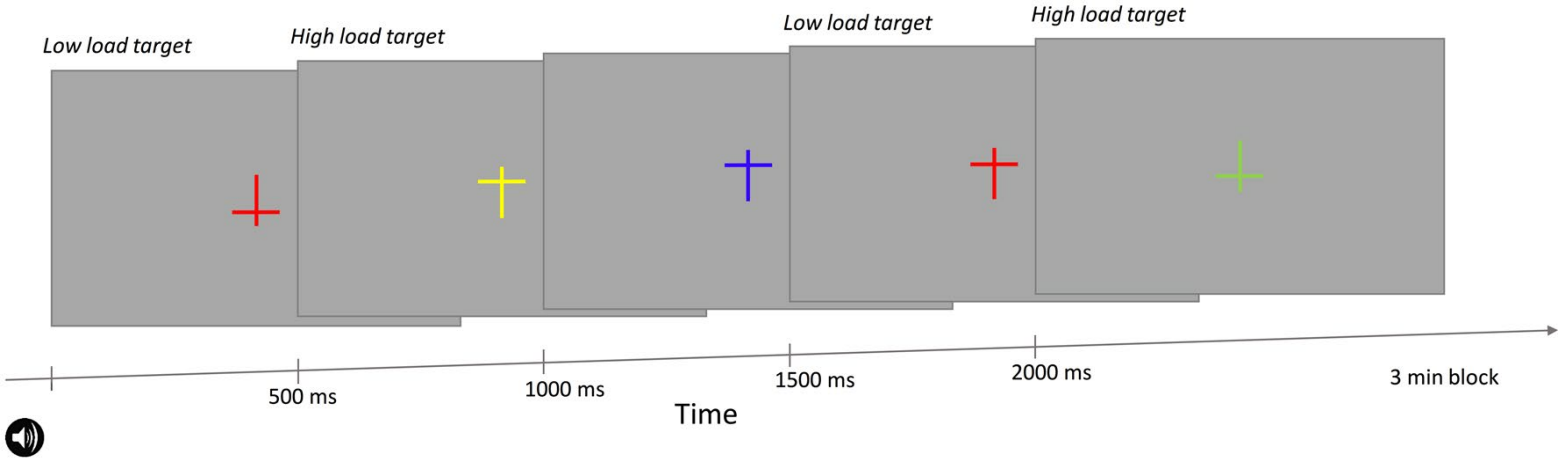
Schematic depiction of the visual task in Study 1. Low and high load involved identical visual stimuli and differed only in which stimuli were designated as the target stimuli (see labels above the visual stimuli). The amplitude‐modulated tone started 200 ms before block onset

Figure 1 illustrates the visual detection task that was modeled after the task in Parks et al. (2011). On each 500‐ms trial, a cross was shown in the center of the screen for 100 ms. Crosses varied in color (red, blue, yellow, green, and violet) and orientation (upright and inverted). The size of the crosses was 3.2° × 3.2° (in visual degrees). In *low load*, subjects responded to any red cross (upright or inverted). In *high load*, subjects responded to upright yellow and inverted green crosses (i.e., conjunction search). Subjects were instructed to press the spacebar on a keyboard as quickly as possible when they detected a target.

Subjects performed eight blocks of the task. Visual load (low or high) alternated over blocks. The initial load level was determined randomly for each subject. Each block consisted of 360 trials and lasted slightly longer than 3 min (360 × 0.5 s = 3 min 3.5 s). The target was presented in 20% of the 360 trials (i.e., 72 targets and 288 nontargets). All combinations of color and orientation were presented equally often and in pseudo‐random order over trials. Each target trial was separated from the next by two to six nontarget trials. At the beginning of each block, seven additional nontarget trials were included so as to avoid presenting a target early on in the block. The amplitude‐modulated tone started 200 ms before each block and was played continuously until the end of the block. Subjects were instructed to ignore the tone while responding to the target crosses.

#### 2.3.2 Working memory capacity task

After the visual task, subjects completed a working memory capacity test (OSPAN, Conway et al., 2005) by Py‐Span‐task software (von der Malsburg, 2015). The task was to remember the sequences of letters. After each letter was displayed for 1 s, a mathematical expression—for example, (8 − 2) × 4 = 28—was shown (as a distractor), and subjects had to read it out loud and judge its correctness. The expression was presented until subjects responded on a keyboard by pressing the letter *c* for correct or *i* for incorrect, or until time ran out (the time limit was adjusted for each subject during a practice session). After each letter sequence, subjects were instructed to type in the letters in their correct order on a keyboard. Sequence length varied between two and six letters, and each sequence length was presented three times. Over trials, the order of the sequence lengths was randomized. For each sequence, the proportion of correctly recalled letters in the correct positions was computed. The partial credit unit (PCU) score was calculated as the mean proportion across all sequences.

### 2.4 EEG recording

The EEG data were recorded from six electrodes at standard 10/20 positions (Fpz, Fz, FCz, Cz, P9, and P10) and from two additional electrodes (at the tip of the nose and on the cheek) with an ActiveTwo BioSemi system (BioSemi, Amsterdam, Netherlands). Fpz, Fz, FCz, Cz, P9, and P10 were recorded with pin electrodes in a 64‐electrode EEG cap. Data from the tip of the nose and the cheek were recorded with flat electrodes attached with adhesive disks. Because the left and right mastoids (M1 and M2) were not available in the EEG cap, we used the nearby positions P9 and P10 for convenience. Two additional, system‐specific positions were recorded with pin electrodes in the EEG cap (https://www.biosemi.com/faq/cms&drl.htm). Data were sampled at 1,024 Hz and filtered with a software high‐pass filter at 0.1 Hz (Butterworth 4th degree two‐pass filter) and a hardware low‐pass filter at 208 Hz.

### 2.5 EEG analysis

#### 2.5.1 Preprocessing

For each block, the first 3 min of auditory stimulation were divided into 115 epochs with a duration of 1.5625 s (the first epoch started with tone onset). At this epoch length, one cycle of the frequency of interest (40.96 Hz) could occur exactly 64 times (1/40.96 × 64 = 1.5625). Also, this epoch length allows satisfactory frequency resolution when the signal is transformed into the frequency spectrum (Δ*f* = 0.64 Hz). Tone onset was identified with a Cedrus StimTracker (Cedrus Corporation, San Pedro, CA, United States). Fpz, Fz, FCz, Cz, and the mastoids (M1 and M2) were re‐referenced to the tip of the nose, and Fpz was also re‐referenced to the cheek electrode to detect vertical and horizontal eye movements.

For each subject, amplitude ranges (i.e., max minus min) within individual epochs were extracted, and the distribution of these ranges was visually inspected to exclude apparent extreme values. Cutoffs were adjusted individually to retain as many trials as possible while reducing the potential effects of extreme values. Because epochs were long (1.56 s) and consecutive epochs in a block covered 3 min of continuous data, eye blinks were inevitable and were not necessarily regarded as extreme values, unless a subject had only a few of them. Notably, because the frequency of interest (40.96 Hz) is much higher than that of eye blinks (<3 Hz), ASSRs should be unaffected by eye blinks. Artifact rejection was unbiased because it was blind to the load level of individual epochs (Keil et al., 2014). After artifact rejection, five subjects had fewer than 70% of epochs left in at least one of the eight blocks. Because 70% was the permitted minimum according to our preregistration, these subjects were excluded from the preregistered analyses. However, they were included in the analyses reported below because their data had no effect on the overall results.

#### 2.5.2 ASSR

For each subject, a mean waveform was computed across all epochs, separately for each of the eight blocks, and each preregistered electrode (Fz and FCz). For the larger sample (*N* = 43) with a lenient artifact rejection, the minimum number of epochs for any block was 104 (i.e., 90.4% of 115 epochs). Each of these 16 mean waveforms was converted into amplitude spectra with a fast Fourier transform. Originally, we preregistered to compute the signal‐to‐noise ratio (SNR) of amplitudes to obtain a single measure that would capture the strength of the signal relative to the noise. However, during data analysis, we realized that amplitude SNR was not the best measure of the difference between signal and noise (as explained in the Supporting Information). To obtain a single measure of the difference between signal and noise, we computed the amplitude difference of signal minus noise (SmN), defined as the amplitude at 40.96 Hz minus the mean amplitude across the neighboring 20 frequencies (ten on each side but omitting two immediate neighbors).

The phase was derived from complex components of the Fourier transform applied to each individual epoch in each condition. The ITC represents the phase coherence among the individual epochs in each condition and can range between 0 (no coherence) and 1 (perfect coherence). For consistency, we report ITC SmN (rather than the ITC). Note that the results for other measures (S, N, and SNR) are available as Supporting Information. For simplicity, we use the terms *amplitude* and *ITC* below to refer to the SmN measures of ASSRs.

#### 2.5.3 Visual P3

Even though we did not preregister to measure the visual P3, we decided to explore it in Study 1 as a manipulation check for the effect of visual load. We computed ERPs for targets and for nontargets, separately for each load condition. The mean number of epochs was at least 64.9 (90.1% of 72) for targets and 255.1 (88.6% of 288) for nontargets in any condition and block. Epochs were extracted from 100 ms before letter onset to 500 ms after letter onset. Each epoch was baseline‐corrected by subtracting the mean amplitude of the 100‐ms interval before letter onset. The data were re‐referenced to the tip of the nose. Fpz was also referenced to the cheek to detect vertical and horizontal eye movements. For each subject, the distribution of epochs in terms of their amplitude ranges (i.e., max minus min within each epoch) was visually inspected, and outlying epochs were removed. Cutoffs were adjusted individually to retain as many trials as possible while reducing the potential effects of outliers. Inspection was blind to the load and target condition of individual trials. The data were low‐pass filtered at 30 Hz for plotting purposes but not for the analyses. To capture the visual P3, mean amplitudes were extracted between 300 and 400 ms across electrode Cz after the onset of the letters. The visual P3 was the difference between targets and nontargets.

### 2.6 Statistical analysis

To evaluate the evidence for or against the alternative hypothesis, we computed the *BF* from Bayesian one‐sample *t* tests of difference scores. Unlike ANOVAs, *t* tests can be used to address specific, informative questions with contrast analyses (Wiens & Nilsson, 2017). In contrast to null hypothesis significance testing, Bayesian hypothesis testing allows one to distinguish among results that support the alternative hypothesis, support the null hypothesis, or are inconclusive (Dienes, 2008, 2016; Wagenmakers, Love, et al., 2018; Wagenmakers, Marsman, et al., 2018; Wiens & Nilsson, 2017). The *BF* compares the likelihood of the data given an alternative hypothesis with the likelihood of the data given the null hypothesis. For example, a *BF*_01_ = 3 indicates that the data are three times more likely under the null hypothesis than under the alternative hypothesis. In contrast, a *BF*_10_ = 3 indicates that the data are three times more likely under the alternative hypothesis than the null hypothesis. Although the *BF* is a continuous measure, we adopted an interpretation scheme in which 3 < *BF* < 10 is considered moderate evidence, 10 < *BF* < 30 is considered strong evidence, 30 < *BF* < 100 is considered very strong evidence, and 100 < *BF* is considered extreme evidence (Wagenmakers, Love, et al., 2018).

We hypothesized that for ASSRs, amplitude and ITC would be smaller during high visual load than during low visual load. Difference scores for amplitude and ITC were obtained by subtracting the values in the high load condition from the values in the low load condition (i.e., low minus high). We modeled the alternative hypothesis (prior) as a uniform distribution from −1 to +1. To capture the possible lower and upper limits of a load effect, we used a wide, two‐tailed alternative hypothesis, as recommended (Dienes, 2014). The null hypothesis was that there would be no differences between the loads. We computed the *BF* with Aladins R scripts (Wiens, 2017). We also hypothesized that the difference scores (low minus high load) would be correlated with WMC scores (but we did not preregister a particular prior). For all mean differences and correlations, we computed 95% confidence intervals to facilitate an estimation approach (Wiens & Nilsson, 2017).

Three exploratory analyses addressed whether the effect of the load varied over time. First, a repeated‐measures ANOVA of load (low vs. high) by block (4 levels) examined whether the load effect varied over blocks (in general or with a linear trend). Second, a repeated‐measures ANOVA of load by minute (1 to 3) examined whether the load effect varied over the 3 min within a block (in general or with a linear trend).

Third, a time‐frequency analysis examined whether the 40‐Hz signal changed periodically with the onset of the visual stimuli, which were shown every 500 ms (i.e., at 2 Hz). If the onsets of the visual stimuli affect the 40‐Hz ASSRs, then the 40‐Hz signal should change at 2 Hz. To detect this change at 2 Hz, epoch length was 10 s (i.e., each epoch contained 20 visual onsets). SmN was defined as the difference between the 2‐Hz signal and the noise, which was defined by surrounding frequencies (10 on each side of 2 Hz, excluding the two nearest neighbors).

A potential concern in this analysis is that the 40‐Hz ASSRs might be confounded by indirect visual effects on the same electrodes as used for ASSRs. That is, the electrodes that were used to record ASSRs might pick up unrelated ERP activity from visual onsets (similarly as for eye blinks). However, because ASSRs are recorded at 40 Hz whereas visual events occur at 2 Hz, an analysis of only the 40‐Hz response should already remove confounding effects of the visual events at 2 Hz (because the frequency is much lower, as for eye blinks). Accordingly, any 2‐Hz activity within the 40‐Hz ASSRs suggests that ASSRs are affected by the visual onsets.

In these exploratory analyses of time, the results of the repeated‐measures ANOVAs are reported from a null hypothesis significance perspective (i.e., *p* values) and from a Bayesian perspective (i.e., *BF* with a Cauchy prior, *r* = .5).

## 3 RESULTS

Table 1 shows the means and 95% confidence intervals of the behavioral and electrophysiological variables for low and high load and for the difference of low minus high load.

**TABLE 1:** Means and 95% confidence intervals for all relevant behavioral and physiological variables, separately for low load, high load, and their difference (low minus high load) in Study 1 (*N* = 43)

Variable	Mean	LL	UL
*d′* low	4.324	4.103	4.545
*d′* high	2.607	2.413	2.8
*d*′ low − high	1.718	1.543	1.893
RT low (ms)	377.807	365.814	389.799
RT high (ms)	505.242	490.861	519.623
RT low − high (ms)	−127.436	−138.628	−116.244
pcu	0.721	0.669	0.774
Amp SmN low (µV)	0.191	0.16	0.222
Amp SmN high (µV)	0.184	0.153	0.215
Amp SmN low − high (µV)	0.006	−0.004	0.017
ITC SmN low	0.309	0.261	0.357
ITC SmN high	0.291	0.241	0.34
ITC SmN low − high	0.019	0	0.038
Visual P3 low (µV)	8.704	6.985	10.423
Visual P3 high (µV)	2.683	1.308	4.058
Visual P3 low − high (µV)	6.02	4.834	7.207

Abbreviations: Amp, amplitude; ITC, intertrial phase coherence; pcu, partial‐credit unit score of working memory capacity; RT, reaction time; SmN, signal minus noise.

### 3.1 Manipulation check of load

#### 3.1.1 Behavioral results

Table 1 shows the mean reaction time to targets and the mean signal‐detection index *d′*, which measures detection ability on the basis of hits and false alarms. Although the definition of hits was not preregistered, they were defined as responses made between 200 and 1,000 ms after visual target onset, whereas false alarms were defined as responses made at other times. Subjects performed better (*d′ M*_diff_ = 1.72, 95% CI [1.54, 1.89]) and were faster (RT *M*_diff_ = 127 ms, 95% CI [116, 139]) during low load than high load. These results confirmed that low load was easier than high load.

#### 3.1.2 Visual P3

**FIGURE 2:**
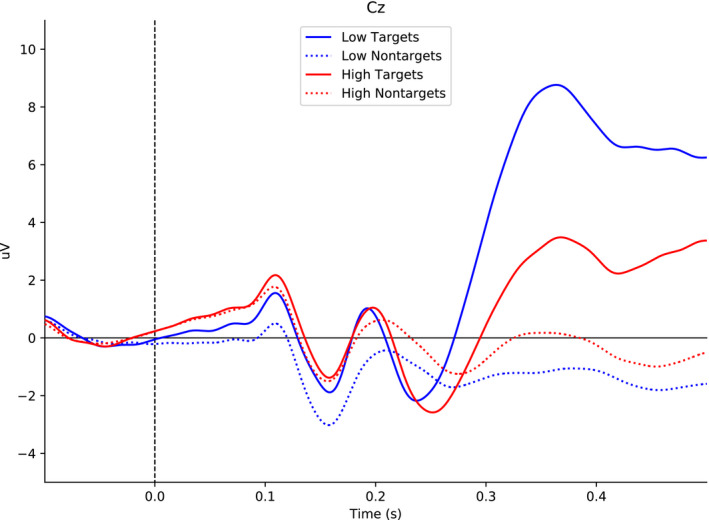
Grand mean ERPs at Cz to targets and nontargets for low and high load in Study 1 (*N* = 43). The visual P3 was extracted from the interval between 300 and 400 ms after stimulus onset. In the figure, the data were low‐pass filtered at 30 Hz

Figure 2 shows the grand mean ERPs for visual targets and nontargets, separately for low and high load. Results showed that the visual P3 (i.e., targets minus nontargets) was larger in low than in high load (*M*_diff_ = 6.02 µV, 95% CI [4.83, 7.21]). This result suggests that low load was easier than high load.

### 3.2 Amplitude

**FIGURE 3:**
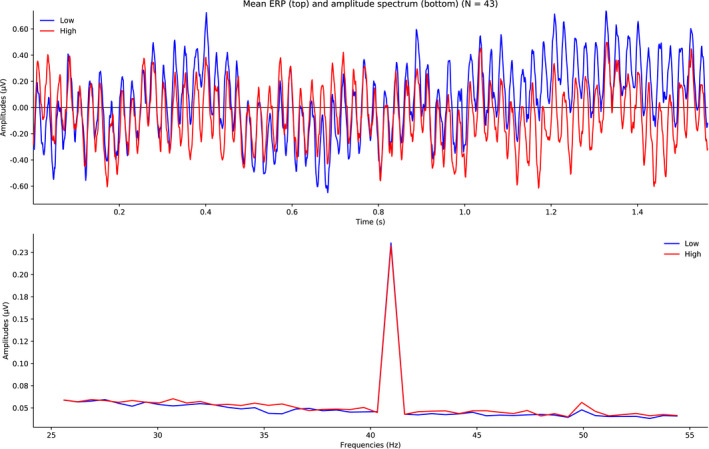
Grand mean ERPs (top) and mean amplitude spectra (bottom) averaged across all blocks and electrodes (Fz, FCz) for low and high load in Study 1 (*N* = 43)

Figure 3 shows the mean ERPs (top panel) and mean amplitude spectra (bottom panel) averaged across electrodes (Fz and FCz) for both low and high load. As shown in Table 1, the amplitude of the frequency of interest (40.96 Hz) was larger than the amplitudes of the neighboring frequencies; thus, the SmN was relatively large (≈ 0.19 µV). In both conditions, the signal amplitude was large (≈ 0.24 µV) whereas the noise amplitude was very low (≈ 0.05 µV). Critically, the amplitude SmN did not differ between the load levels (SmN difference = 0.006 µV, 95% CI [−0.004, 0.017]). In support, the *BF* provided very strong evidence for the null hypothesis (*BF*_01_ = 72.4) when the alternative hypothesis was modeled as a uniform distribution from −1 to +1, as preregistered. Although this alternative hypothesis states that load effects could be in either direction, another reasonable alternative hypothesis is that the amplitude SmN should be smaller in high load than in low load. This one‐tailed hypothesis (between 0 and +1) provided very strong evidence for the null (*BF*_01_ = 40.1).

### 3.3 Intertrial phase coherence

As shown in Table 1, ITC at the frequency of interest (40.96 Hz) was larger than ITC at the neighboring frequencies; thus, the SmN was relatively large (≈ 0.30). In both conditions, the signal was large (≈ 0.38) whereas the noise was very low (≈ 0.08). Critically, the ITC SmN did not differ between the load levels (SmN difference = 0.019, 95% CI [0, 0.038]. In support, the *BF* provided strong evidence for the null hypothesis (*BF*_01_ = 12.1) with the alternative hypothesis modeled as a uniform distribution from −1 to +1, as preregistered. Even when the alternative hypothesis was one‐tailed (between 0 and 1), results provided moderate support for the null hypothesis (*BF*_01_ = 6.2).

### 3.4 Working memory capacity

Working memory capacity did not correlate with the effect of load on the amplitude (*r* = .008, 95% CI [−0.284, 0.291], *BF*_01_ = 5.3) or with the effect of load on the ITC (*r* = −.020, 95% CI [−0.313, 0.272], *BF*_01_ = 5.2). In these analyses, the *BF* and the credible intervals were calculated with the alternative hypothesis modeled as a flat prior (*β* = 1).

### 3.5 Effect of time

In a recent study (Yokota et al., 2017), the load effect on ITC varied with time on task: The ITC was higher during low load than during high load only in the first half of the experiment. To determine whether the load effect varied over time, we conducted exploratory ANOVAs of load (2 levels: low vs. high) by block (4 levels), as shown in the Supporting Information. In these 2 × 4 ANOVAs, the overall interaction (with 3 *df*s) of load by block and the contrast interaction (with 1 *df*) of load by the linear trend of the block were not significant for either amplitude or ITC (all *p* > .68). Exploratory Bayesian ANOVAs (with a Cauchy prior, *r* = .5) suggested strong evidence that a model with only the main effects (i.e., load and block) explained the data better than a model that also included the interaction, both for amplitude and for ITC (both *BF*_01_ > 21). Additionally, to test whether the load effect varied within a block of 3 min, we divided each block into three miniblocks of 1 min each, averaged each minute across the four blocks of load, and conducted exploratory 2 × 3 ANOVAs of load (low vs. high) by minute (1st, 2nd, 3rd). The overall interaction (with 2 *df*s) of load by minute and the contrast interaction (with 1 *df*) of load by the linear trend of minute were not significant for either amplitude or ITC (all *p* > .33). In support, Bayesian analyses suggested strong evidence for a model without the interaction (both *BF*_01_ > 10). Last, the time‐frequency analysis for a 2‐Hz signal within the 40‐Hz ASSRs provided moderate evidence for no effect of load (*p* = .626, *BF*_01_ = 4.1).

## 4 STUDY 2

Study 2 was a follow‐up to Study 1. Because the method and analyses were identical to those of Study 1 in many aspects, only deviations are listed below.

### 4.1 Method

All Supporting Information is available at a university depository (Wiens & Szychowska, 2020). This material includes raw data, scripts, and results of main and supplementary analyses.

The method and analyses were preregistered before any data were collected (https://doi.org/10.17605/OSF.IO/JVMFD). Forty‐seven subjects were recruited from local universities and through online billboards in Stockholm, Sweden. Two subjects had to be excluded because of the failure of the StimTracker. The complete sample for analyses of ASSRs consisted of 45 subjects (age *M* = 27.2, *SD* = 4.77, 42 right‐handed, 21 male). Because the results for ASSRs in Study 1 suggested that an exclusion criterion of less than 70% of epochs for any block was too strict, this exclusion criterion was not used in Study 2. Thus, all subjects were retained for analyses of ASSRs.

Because of photodiode failure, the onset of the visual stimuli could not be identified for three subjects. Therefore, the results for the visual P3 and for the visual onsets (at 2‐Hz) on ASSRs are based on only 42 subjects. Although we preregistered that subjects would perform a working memory test at the end of the whole experiment, the first three subjects reported feeling tired after the visual task. Thus, after the third subject, the working memory task was administered before the visual task. The first three subjects were excluded from the analyses involving WMC. Two more subjects were excluded because of a program failure. Thus, the results for WMC are based only on 40 subjects. Although we preregistered that data collection would finish by the end of June 2019, we continued collecting data after that date until we reached *BF* > 3.

### 4.2 Procedure

Subjects performed a visual search task on a sequence of letters of varying color, name, and capitalization while ignoring a tone played continuously in the background. After each block, subjects rated the subjective workload of the task. Before the experiment, subjects performed a WMC test.

#### 4.2.1 Visual detection task

**FIGURE 4:**
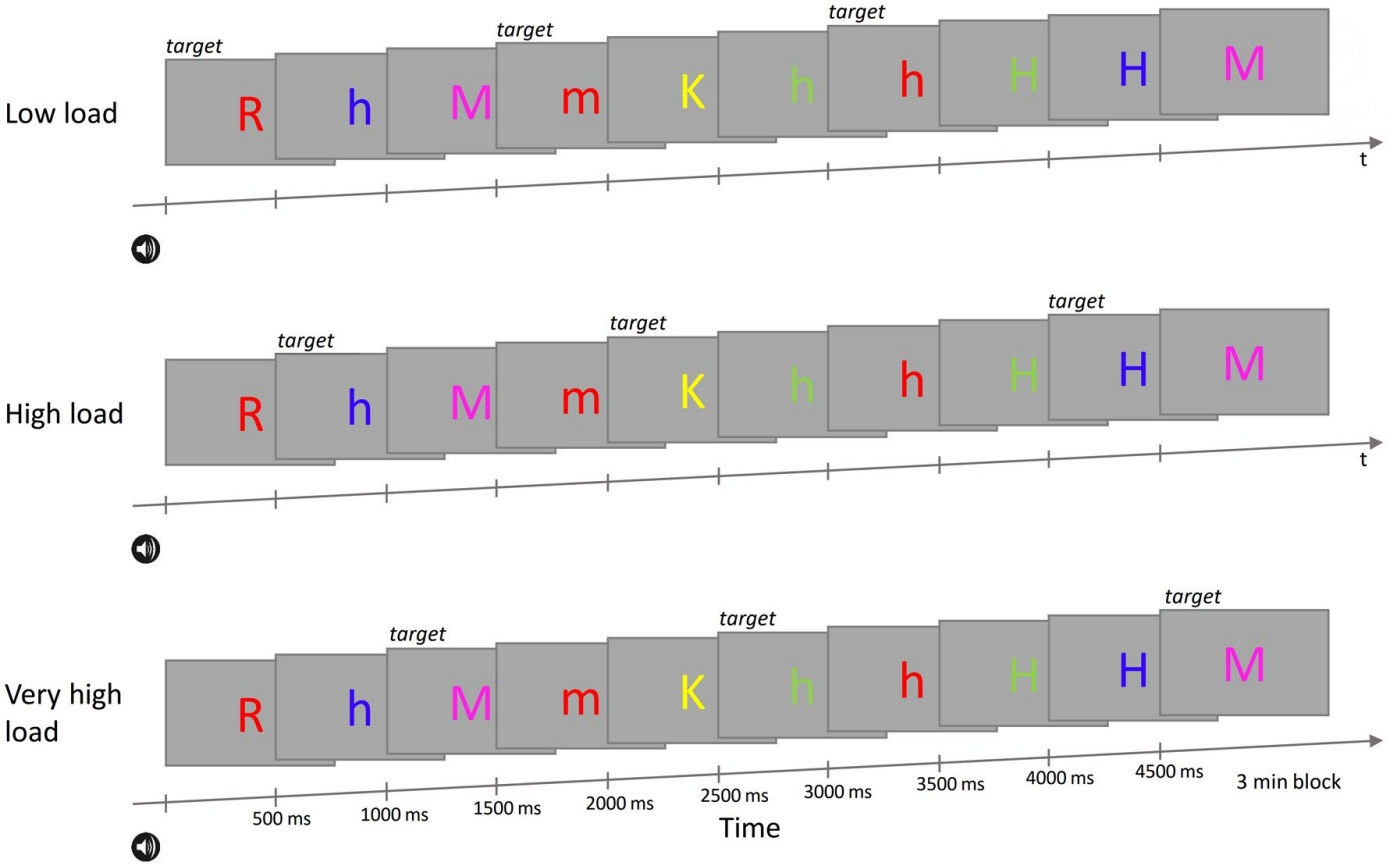
Schematic depiction of the visual task in Study 2. Low, high, and very high load involved similar visual stimuli but differed in which stimuli were designated as the target stimuli (see labels above the visual stimuli). The amplitude‐modulated tone started 200 ms before block onset

The visual stimuli were letters that differed in name (K, R, M, or H), color (red, blue, green, yellow, or violet), and capitalization (uppercase or lowercase). Each block had one of the four conditions: no load, low load, high load, and very high load. Figure 4 illustrates the low load, high load, and very high load conditions in the visual search task. In *no load*, there was no target, and subjects passively viewed the letters presented on the screen. In *low load*, targets were letters of a particular color regardless of name and capitalization. In *high load*, targets were particular name‐color combinations regardless of capitalization; for example, yellow K or k, and blue H or h. Thus, low load and high load resembled the conditions in Study 1. In *very high load*, targets were combinations of name, color, and capitalization; for example, green h and violet M. Subjects were instructed to ignore the task‐irrelevant background tone and to perform the visual task; that is, either to respond to target letters as quickly and accurately as possible (in the three load conditions) or to view the letters passively (in the no load condition). Note that the condition labels used here for high and very high load differ from those in the preregistration: The label *high* corresponds to the label *medium* in the preregistration, and the label *very high* corresponds to the label *high* in the preregistration. The labels were changed to keep the labeling consistent between the studies.

Subjects performed 16 blocks of the task. Each condition was administered four times. For each subject, the order was randomly determined within a set of four conditions. For each subject, targets in the different conditions were chosen pseudo‐randomly to avoid conflicts. For example, if red was the target in low load, no other condition for that subject had a red target. Also, if for one subject, yellow letters were assigned as targets in low load, then for another subject, blue letters were assigned as targets in low load. In the different conditions, the visual stimuli were physically identical but occurred in different proportions (to retain a constant proportion of targets). Thus, any differences in the ASSRs are unlikely to be due to physical differences in the load conditions. The targets and nontargets were drawn randomly without replacement from target and nontarget pools, separately for each subject and condition.

#### 4.2.2 Self‐reported workload

After each block, subjects rated the experienced workload during the block by responding to the NASA‐Task Load Index (NASA‐TLX) (Hart & Staveland, 1988). Participants rated mental demand, physical demand, temporal demand, performance, effort, and frustration. The NASA‐TLX's original rating scale was replaced with the Borg CR100 scale (Borg & Borg, 2002). One advantage of the Borg CR100 scale is that it uses explicit verbal anchors. For example, 100 is represented by *maximal*, and 50 is represented by *strong* and *heavy*. Another advantage is that the scale allows subjects to use values above 100, which avoids ceiling effects.

### 4.3 EEG

The EEG data were recorded as in Study 1 except that we recorded CPz and Pz (instead of P9 and P10, to improve measurement of the visual P3). Electrodes CPz and Pz were re‐referenced to the tip of the nose. All subjects were retained because we did not use the same strict exclusion criterion (of at least 70% epochs in each block) used in Study 1. For ASSRs, the minimum number of epochs for any block was 105 (i.e., 91.3% of 115 trials). To capture the visual P3, mean amplitudes were extracted between 300 and 400 ms after the onset of the letters across electrodes Cz, CPz, and Pz. For the low, high, and very high load conditions, the visual P3 was the difference between targets and nontargets. For the visual P3, the mean number of epochs was at least 64.5 (89.6% of 72) for targets and 247.6 (86.0% of 288) for nontargets in any condition and block.

### 4.4 Statistical analysis

We preregistered three hypotheses: The difference between very high load and low load captures the effect of load, the difference between low load and no load captures the effect of task, and the difference between very high load and no load captures the combined effect of load and task. However, as described below, results suggested that high load was more difficult than the very high load. Therefore, we used high load rather than very high load to test the hypotheses.

## 5 RESULTS

The main results are shown in figures. For completeness, the exact means and 95% confidence intervals of the behavioral and electrophysiological variables for low, high, and very high load, and for the relevant difference scores are available as Supporting Information (Wiens & Szychowska, 2020).

### 5.1 Manipulation check of load

#### 5.1.1 Behavioral results

**FIGURE 5:**
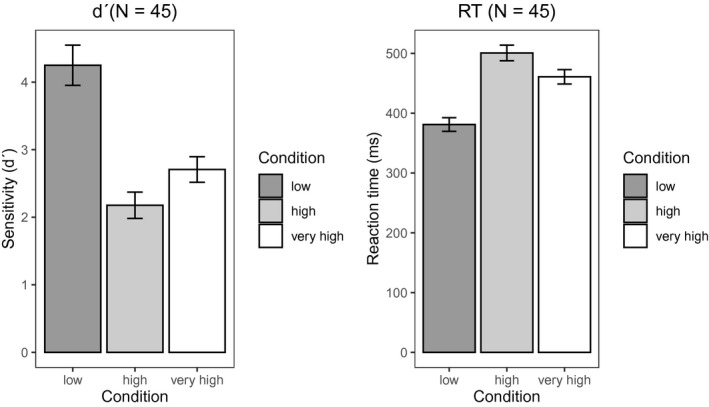
Mean *d′* (left) and mean reaction times (right) averaged across all blocks for low, high, and very high load in Study 2 (*N* = 45)

Figure 5 shows the mean signal‐detection index *d′* and mean reaction times to targets in low, high, and very high load. The figures suggested that subjects performed best and were fastest during low load, and they performed worst and were slowest during high load. Surprisingly, the very high load was not the most difficult one. This pattern of results was supported by the 95% CIs of the difference scores between conditions (see Supporting Information). Results were similar for visual P3 and subjective ratings of workload (see below).

#### 5.1.2 Visual P3

**FIGURE 6:**
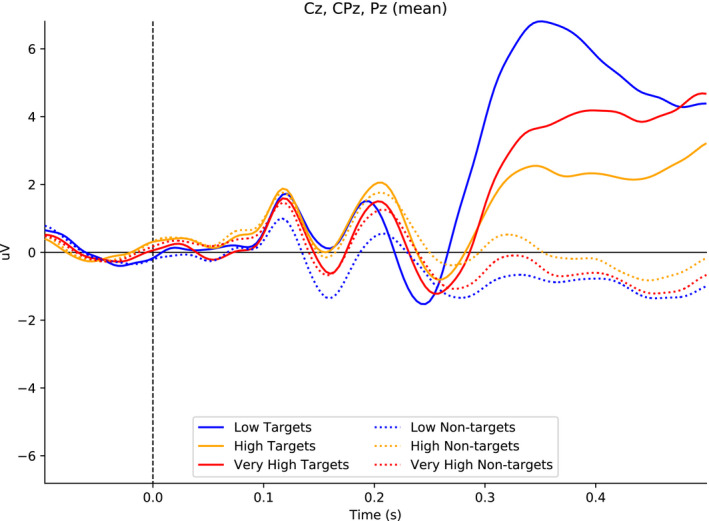
Grand mean ERPs across Cz, CPz, and Pz to targets and nontargets for low, high, and very high load in Study 2 (*N* = 42). The visual P3 was extracted from the interval between 300 and 400 ms after the stimulus onset. In the figure, the data were low‐pass filtered at 30 Hz

As suggested in Figure 6, the visual P3 was largest in low load (*M* = 6.9 µV), smaller in very high load (*M* = 3.9 µV), and smallest in high load (*M* = 2.1 µV). These results suggest that high load was more difficult than both low load and very high load. In support, low minus high *M*_diff_ = 4.8 µV, 95% CI [3.5, 6.1], and very high minus high *M*_diff_ = 1.8 µV, 95% CI [1.0, 2.5].

#### 5.1.3 Workload ratings

**FIGURE 7:**
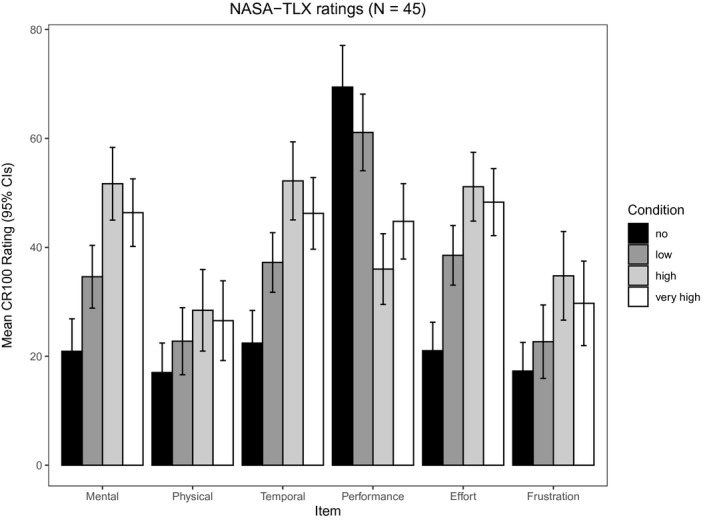
Mean ratings of self‐reported workload (NASA‐TLX) for no, low, high, and very high load in Study 2 (*N* = 45)

As shown in Figure 7, among the four conditions, high load had the highest ratings on mental demand, physical demand, temporal demand, effort, and frustration. In contrast, it had the lowest ratings on performance. Because high load was clearly more difficult than very high load (see Supporting Information), we deviated from the preregistration and used high load (rather than very high load) to capture the effects of load.

### 5.2 Amplitude

**FIGURE 8:**
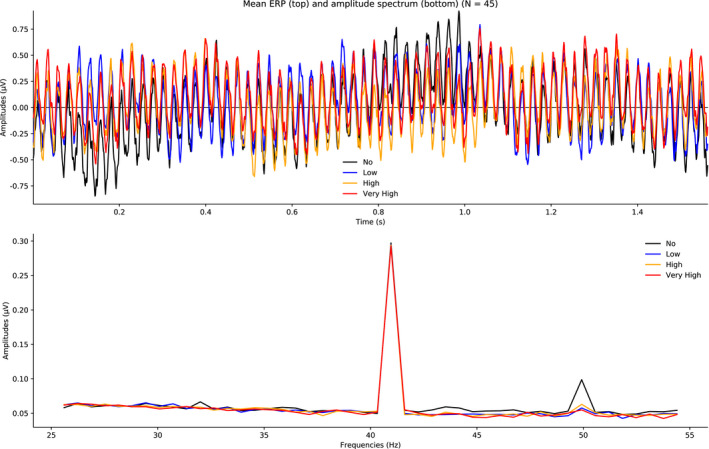
Grand mean ERPs (top) and mean amplitude spectra (bottom) averaged across all blocks and electrodes (Fz, FCz) for no, low, high, and very high load in Study 2 (*N* = 45)

**FIGURE 9:**
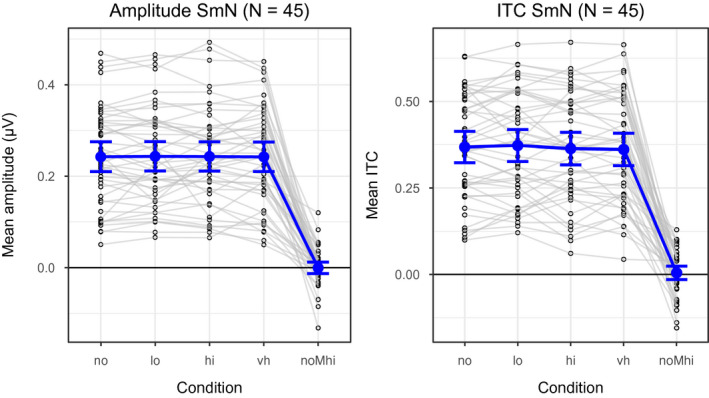
Means and 95% CIs of signal minus noise (SmN) measures of amplitude (left) and intertrial coherence (ITC, right) for all conditions and for the combined effect of load and task (no load minus high load) in Study 2 (*N* = 45). Individual subjects' values are plotted as circles, and the gray lines connect all values for each individual. lo = low, hi = high, vh = high load, and noMhi = no minus high

Figure 8 shows mean ERPs (top panel) and mean amplitude spectra (bottom panel) averaged across two electrodes (Fz and FCz) for the four loads. The amplitude of the frequency of interest (40.96 Hz) was substantially larger than the amplitudes of the neighboring frequencies; for all conditions, the SmN was large, SmN ≈ 0.24 µV. Critically, as shown in Figure 9, the SmN did not differ among the conditions (all SmN differences < 0.002). In support, the *BF*s (see Table 2) provided extreme evidence for the null hypothesis (*BF*_01_ > 123) for all relevant comparisons (e.g., no vs. high load) with the alternative hypothesis modeled as a uniform distribution from −1 to +1. Similar results were obtained with a one‐tailed hypothesis (0 to +1).

**TABLE 2:** Mean differences and Bayes factors (*BF*s) for signal minus noise (SmN) measures of amplitude and intertrial phase coherence (ITC) for the effects of load (low minus high), task (no minus low), and load and task combination (no minus high) in Study 2. *BF*_01_ captures the strength of the evidence for the null hypothesis relative to the alternative hypothesis. The alternative hypothesis was modeled as a uniform distribution that ranged from −1 to +1 (H1), 0 to +1 (H2), or 0 to +0.2 (H3)

Variable	Mean	Bayes factor (*BF*_01_)		
		H1	H2	H3
Amp no − low	−0.0009	144.1	152.2	33.5
Amp no − high	−0.0004	127.0	125.7	27.2
Amp low − high	0.0004	123.8	110.8	23.7
ITC no − low	−0.0044	84.5	135.0	29.4
ITC no − high	0.0045	73.5	52.7	10.9
ITC low − high	0.0090	53.4	31.5	6.4

Abbreviation: Amp, amplitude.

### 5.3 Intertrial phase coherence

The ITC at the frequency of interest (40.96 Hz) was larger than the ITC at the neighboring frequencies (SmN ≈ 0.37). Critically, as illustrated in Figure 9, the ITC SmN did not differ between the conditions (−0.005 < SmN difference < 0.012). In support, the *BF*s (see Table 2) provided very strong evidence for the null hypothesis (*BF*_01_ > 53) for all relevant comparisons (e.g., no vs. high load) with the alternative hypothesis modeled as a uniform distribution from −1 to +1. Similar results were obtained with a one‐tailed hypothesis (0 to +1).

### 5.4 Working memory capacity

Working memory capacity did not correlate with any of the effects (load, task, combined) in terms of amplitude (−0.13 < *r* < .12, all *BF*_01_ > 3.7) or ITC (−0.08 < *r* < .15, all *BF*_01_ > 3.4). In these analyses, the *BF* was calculated with the alternative hypothesis modeled as a flat prior (*β* = 1).

### 5.5 Effect of time

ANOVAs (2 × 4) were used to test the combined effect of load and task (2 levels: no vs. high) on the amplitude and ITC by block (4 levels). The overall interaction (with 3 *df*s) of load by block and the contrast interaction (with 1 *df*) of load by the linear trend of the block were not significant for either amplitude or ITC (all *p* > .32). Exploratory Bayesian ANOVAs suggested strong evidence that a model with only the main effects (i.e., load and block) explained the data better than a model that also included the interaction, both for amplitude and for ITC (both *BF*_01_ > 13). Additionally, ANOVAs of load (no vs. high) by minute (1st, 2nd, 3rd) were used to test the combined effect of load and task (no vs. high) within a block. The overall interaction (with 2 *df*s) of the combined effect by minute and the contrast interaction (with 1 *df*) of the combined effect by the linear trend of minute were not significant for either amplitude or ITC (all *p* > .27). Bayesian ANOVAs also favored a model without the interaction (both *BF*_01_ > 7). Last, the time‐frequency analysis for a 2‐Hz signal within the 40‐Hz ASSRs provided strong evidence for no effect of load (*p* = .449, *BF*_01_ = 11.8).

## 6 GENERAL DISCUSSION

We recorded 40‐Hz ASSRs to task‐irrelevant tones while subjects performed a visual task with either low or high load (Study 1), or a visual task with no, low, high, or very high load (Study 2). The combined results provided strong to extreme evidence for no effect of visual load (low vs. high load) on 40‐Hz ASSRs. Also, the results of Study 2 provided very strong to extreme evidence for no effect of task (no vs. low load) and for no effect of a combination of task and load (no vs. high load) on 40‐Hz ASSRs. Exploratory analyses of time provided moderate to strong evidence that these results did not vary over blocks, within blocks, or in terms of activity that was synchronized to the visual onsets (at 2 Hz). Further, results from both studies provided moderate support for no correlation between WMC and a load effect on 40‐Hz ASSRs.

### 6.1 High load more difficult than very high load

The purpose of Study 2 to examine the effects of load on 40‐Hz ASSRs by including a stronger task manipulation than in Study 1. In Study 1, visual targets were crosses that varied in terms of color and orientation. For Study 2, we thought that adding a third dimension would make the task more difficult. Accordingly, whereas visual targets in high load varied only by color and name, targets in very high load varied by color, name, and capitalization. In high load, example targets are yellow K and k, and blue H and h. In very high load, example targets are green h and violet M. Results clearly showed that our prediction was incorrect: High load was more difficult than very high load. Specifically, compared to very high load, high load was characterized by poor behavioral performance (*d*′ and RT), small visual P3, and high ratings of workload. Consistent with these findings, previous research suggests that capitalized and noncapitalized letters are treated differently: It is more difficult to recognize that two letters are the same when they differ in capitalization than when they are physically identical (Hellige & Webster, 1981; McDermott, Bavelier, & Green, 2014; Posner & Mitchell, 1967). Accordingly, high load might have been more difficult than very high load because the same letters with different capitalizations were treated as separate items. For example, the targets yellow K and k, and blue H and h in high load were actually treated as four rather than two targets. Thus, high load was more difficult than very high load, which had only two targets (e.g., green h and violet M). Critically, because substantial load effects were observed for high load in Study 2 in terms of task performance, visual P3, and subjective ratings of workload, our misclassification of very high load does not challenge our results with regard to load effects on ASSRs.

Notably, when we compared performance differences between studies (see Supporting Information), *d*′ tended to decrease more strongly from low load to high load in Study 2 than in Study 1 (*M*_diff_ = −0.32, 95% CI [−0.56, −0.07]), whereas effects on reaction time were similar for both studies (*M*_diff_ = −7.58 ms, 95% CI [−22.11, 6.94]). These results suggest that load effects (from low to high) were stronger in Study 2 than in Study 1.

### 6.2 No effect of load, task, or their combination on 40‐Hz ASSRs

Results provided moderate to extreme evidence (12 < *BF*_01_ < 145) that both amplitude and ITC of the 40‐Hz ASSRs were unaffected by load, task, or the combination of load and task (e.g., Table 2 for Study 2). Furthermore, exploratory analyses of time (i.e., block, minute, and activity that was synchronized to the visual onsets) provided moderate to strong evidence (7 < *BF*_01_ < 24) for no interactions with load. The findings of no load effect match those reported by Parks et al. (2011). These authors used a perceptual load manipulation similar to ours.

A possible explanation for the absence of an effect is that this particular load manipulation is not strong enough. However, in our two studies and in the Parks et al. (2011) study, subjects showed large differences in behavioral performance between loads. These findings are strong manipulation checks that high load was substantially more difficult than low load. Furthermore, in other studies that used the same load manipulation and reported load effects on other variables, the load effects on behavioral performance were similar to ours (Jacoby, Hall, & Mattingley, 2012; Kamke et al., 2012; Parks et al., 2011; Schwartz et al., 2005). Last, the type of load used in the present study has been advocated as a prototypical manipulation of perceptual load (Lavie, 2005). Further, whereas previous studies used only behavioral measures as a manipulation check for load, we found substantial load effects on the visual P3 to targets and nontargets and on subjective workload ratings. In terms of mental demand, temporal demand, and effort, subjects rated high load as strong and heavy. Taken together, the arguments that high load was not strong enough are not convincing.

Another potential explanation is that the tone was hard to ignore at about 60 dB SL. However, this sound level is considered comparable to that of average conversations in restaurants and offices (IAC Acoustics, 2020). Also, it appears that no previous study of the effects of visual load on ASSRs has used auditory stimuli with sound levels lower than 60 dB. Nevertheless, we cannot rule out the possibility that at lower sound levels, subjects would be better at ignoring the sounds.

Yet another potential explanation is that although auditory processing was dampened or filtered, we simply missed the auditory processing stage at which the filtering occurred. According to the adaptive filtering model (Giard et al., 2000), the filtering stage varies with the difficulty or load of the visual task. Two situations are thus conceivable: First, if low load is already highly demanding, the filtering may have started before or at the level of wave V coming from the brainstem, or MLRs coming from the auditory cortex (in line with the superposition hypothesis). In this case, high load had no effect because filtering occurred in both low and high load. However, the low load task was a simple color‐detection task (pop‐out effect), and performance was at the ceiling. Nonetheless, we added the condition of no load in Study 2 and found no effect on the 40‐Hz ASSRs without versus with a task. Thus, this explanation is unlikely. Second, high load may dampen auditory processing only after the auditory pathway stage that generates MLRs. Accordingly, if it is assumed that 40‐Hz ASSRs comprise an overlap of wave V from the brainstem and MLR (in line with the superposition hypothesis), the ASSRs in the present research did not differ with load and task because we recorded responses that happen before the filtering occurred. However, because primary auditory cortex is the main generator of 40‐Hz ASSRs (Brugge et al., 2009; Engelien, 2000; Gutschalk, 1999; Herdman, 2002; Pantev, 1996; Ross, Miyazaki, & Fujioka, 2012), this explanation would be inconsistent with findings that load affects activations related to figure‐ground segregation in the auditory cortex, as measured with magnetoencephalography (Molloy, Lavie, & Chait, 2019).

To examine whether attentional filtering can occur earlier or later in the auditory pathway, future studies should use modulation frequencies other than 40 Hz. For faster modulations (e.g., 80 Hz), ASSRs seem to originate mostly from the brainstem (Giraud et al., 2000; Herdman et al., 2002; Kuwada et al., 2002; Luke et al., 2017; Picton et al., 2003; Plourde et al., 1991), whereas, for slower modulations (below 40 Hz), ASSRs seem to originate mostly from the auditory cortex (Giraud et al., 2000; Kuwada et al., 2002; Luke et al., 2017). Accordingly, faster modulations may be informative about earlier stages of the auditory pathway (brainstem), and slower modulations may be informative about later processing stages.

### 6.3 No correlation with WMC

In both studies, we examined a proposed relationship between WMC and the effect of load on auditory distractors (Marsh & Campbell, 2016). We did not predict any particular direction of the correlation because of the inconsistent findings of previous studies (Halin et al., 2015; Sörqvist et al., 2012). The present results provide moderate support for no correlation between WMC and the effect of load and task on ASSRs. As such, the present findings are not consistent with the proposed role of working memory capacity in auditory filtering (Marsh & Campbell, 2016). In contrast, our findings are consistent with recent evidence in a large sample (*N* = 601) for no relationship between WMC and either the changing state effect or the deviation effect in serial recall (Körner, Röer, Buchner, & Bell, 2017).

The present results suggest that visual load has no effect on the amplitude and intertrial coherence of ASSRs to a 40‐Hz amplitude‐modulated tone. This is useful information for clinicians who use ASSRs to test hearing thresholds in individuals who are unable to provide a subjective response (e.g., preverbal children). If ASSRs are unaffected by whether individuals engage in a low or high visual load task or no task, results of clinical audiometry with ASSRs should be unaffected by the directed attention of the tested individuals.

To conclude, the present findings support the robustness of 40‐Hz ASSRs against manipulations of crossmodal attention and, thus, are not consistent with the adaptive filtering model. However, even if the filtering is not adaptive, it can occur at the same stage of the pathway for all load conditions. If so, the current results would be consistent with the early‐filter theory.

## AUTHOR CONTRIBUTIONS

**Malina Szychowska:** Conceptualization; Data curation; Formal analysis; Investigation; Methodology; Project administration; Software; Validation; Visualization; Writing‐original draft; Writing‐review & editing. **Stefan Wiens:** Conceptualization; Data curation; Formal analysis; Funding acquisition; Investigation; Methodology; Project administration; Resources; Software; Supervision; Validation; Visualization; Writing‐original draft; Writing‐review & editing.
